# Switch of Voltage-Gated K^+^ Channel Expression in the Plasma Membrane of Chondrogenic Cells Affects Cytosolic Ca^2+^-Oscillations and Cartilage Formation

**DOI:** 10.1371/journal.pone.0027957

**Published:** 2011-11-21

**Authors:** Zoltan Varga, Tamás Juhász, Csaba Matta, János Fodor, Éva Katona, Adam Bartok, Tamás Oláh, Attila Sebe, László Csernoch, Gyorgy Panyi, Róza Zákány

**Affiliations:** 1 Department of Biophysics and Cell Biology, University of Debrecen Medical and Health Science Center, Debrecen, Hungary; 2 Department of Anatomy, Histology and Embryology, University of Debrecen Medical and Health Science Center, Debrecen, Hungary; 3 Department of Physiology, University of Debrecen Medical and Health Science Center, Debrecen, Hungary; Universidad de Castilla-La Mancha, Spain

## Abstract

**Background:**

Understanding the key elements of signaling of chondroprogenitor cells at the earliest steps of differentiation may substantially improve our opportunities for the application of mesenchymal stem cells in cartilage tissue engineering, which is a promising approach of regenerative therapy of joint diseases. Ion channels, membrane potential and Ca^2+^-signaling are important regulators of cell proliferation and differentiation. Our aim was to identify such plasma membrane ion channels involved in signaling during chondrogenesis, which may serve as specific molecular targets for influencing chondrogenic differentiation and ultimately cartilage formation.

**Methodology/Principal Findings:**

Using patch-clamp, RT-PCR and Western-blot experiments, we found that chondrogenic cells in primary micromass cell cultures obtained from embryonic chicken limb buds expressed voltage-gated Na_V_1.4, K_V_1.1, K_V_1.3 and K_V_4.1 channels, although K_V_1.3 was not detectable in the plasma membrane. Tetrodotoxin (TTX), the inhibitor of Na_V_1.4 channels, had no effect on cartilage formation. In contrast, presence of 20 mM of the K^+^ channel blocker tetraethyl-ammonium (TEA) during the time-window of the final commitment of chondrogenic cells reduced K_V_ currents (to 27±3% of control), cell proliferation (thymidine incorporation: to 39±4.4% of control), expression of cartilage-specific genes and consequently, cartilage formation (metachromasia: to 18.0±6.4% of control) and also depolarized the membrane potential (by 9.3±2.1 mV). High-frequency Ca^2+^-oscillations were also suppressed by 10 mM TEA (confocal microscopy: frequency to 8.5±2.6% of the control). Peak expression of TEA-sensitive K_V_1.1 in the plasma membrane overlapped with this period. Application of TEA to differentiated chondrocytes, mainly expressing the TEA-insensitive K_V_4.1 did not affect cartilage formation.

**Conclusions/Significance:**

These data demonstrate that the differentiation and proliferation of chondrogenic cells depend on rapid Ca^2+^-oscillations, which are modulated by K_V_-driven membrane potential changes. K_V_1.1 function seems especially critical during the final commitment period. We show the critical role of voltage-gated cation channels in the differentiation of non-excitable cells with potential therapeutic use.

## Introduction

Due to the lack of blood supply and the postmitotic nature of fully differentiated adult chondrocytes, articular cartilage has very limited self-repair capability following tissue damage. Recent therapeutic attempts to restore articular cartilage mass and function have focused on regenerative cell-based techniques, including autologous chondrocyte implantation and autologous mesenchymal stem cell transplantation [1], [2]. Both techniques require *ex vivo* expansion of the cells and the phenotype of the cells to be transplanted is extremely sensitive to the culturing environment [3]. Therefore, to tightly control cell proliferation and chondrogenic differentiation, a detailed knowledge of the signal transduction mechanisms involved in these processes is required. Many external stimuli initiate large-scale cellular changes via altering ion channel activities, which in turn manifest in the associated changes in membrane potential and intracellular Ca^2+^ concentration ([Ca^2+^]_i_).

Cyclic changes in [Ca^2+^]_i_ resulting in global events are well documented in excitable cells and are reported to be linked to controlling gene expression [4]. Non-excitable cells, such as endothelial cells [5] and osteoblasts [6] were also shown to display calcium oscillations, where ion channels from both the plasma membrane and from intracellular stores were found to be associated with these phenomena [7]. In particular, such events have been detected in isolated mature articular chondrocytes cultured in agarose constructs [8]. In chicken embryonic chondrogenic cells, we have previously described characteristic changes of the free cytosolic [Ca^2+^]_i_, which was dependent on extracellular Ca^2+^ and was associated with calcineurin activity, as well as evidence for purinergic Ca^2+^-signaling via P2X_4_ receptors. These phenomena were temporally synchronized with chondrocyte differentiation [9], [10].

Signaling pathways that involve changes in [Ca^2+^]_i_ are tightly coupled to the activity of plasma membrane ion channels and consequent changes in the membrane potential. Pathways that use Ca^2+^ as a second messenger necessitate channels that allow Ca^2+^ influx from the extracellular space and most often also employ other channels that stabilize the membrane potential [11]. Signaling during differentiation often brings about changes in the expression of these channels. Membrane potential has been reported among the key regulators of proliferation in a number of cell types, implying that its modulation is required for both G1/S phase and G2/M phase transitions. Depolarization of the membrane through changes in extracellular ion concentration inhibits G1/S progression in several cell types such as lymphocytes, astrocytes, fibroblasts and Schwann cells, suggesting that hyperpolarization is required for the initiation of S phase [12].

Numerous factors influence the membrane potential of cells, among which voltage-gated cation channels have fundamental importance. A wide array of voltage-gated K^+^ (K_V_), Na^+^ (Na_V_) and Ca^2+^ channels is known, characterized by various electrophysiological properties [13]. When the membrane is depolarized, voltage-gated channels open rapidly, allowing ions to flow passively down their electrochemical gradients at near diffusion rates. Voltage-gated channels thus regulate the membrane potential through their two principal functions: voltage sensing and ion conduction, which are accomplished by two distinct, but functionally coupled structures in each subunit or domain [14].

Although ion channels and membrane potential changes have been described earlier in mature chondrocytes isolated from cartilage of various species or cells cultured for longer periods [15]–[23], this is the first report to track the changes in the ion channel expression of chondrogenic cells differentiating to chondrocytes.

In the chondrogenesis model applied, chondroprogenitor mesenchymal cells isolated from limb buds of chicken embryos spontaneously differentiate into chondrocytes predominantly on the second and third culturing days and secrete a considerable amount of cartilage-specific extracellular matrix by the end of the six-day-long culturing period. Progression of cartilage differentiation can easily be followed by the detection of mRNA expression of Sox9, the master transcription factor of cartilage formation [24], [25], as well as by monitoring the expression of cartilage matrix components, such as type II collagen and the cartilage-specific proteoglycan aggrecan.

In the current study, our aim was the investigation of the signal transduction pathways of early chondrogenic differentiation. We succeeded in identifying voltage-gated Na^+^ and K^+^ channels and describing the changes in their expression patterns in chondrogenic cells. We characterized rapid cytosolic calcium oscillations and plasma membrane potential changes, both dependent on the identified TEA-sensitive K_V_-channels, and we observed the connection of these phenomena with the progression of chondrocyte differentiation.

## Results

### TEA-sensitivity of high-frequency Ca^2+^ oscillations changes with differentiation

**Figure 1:**
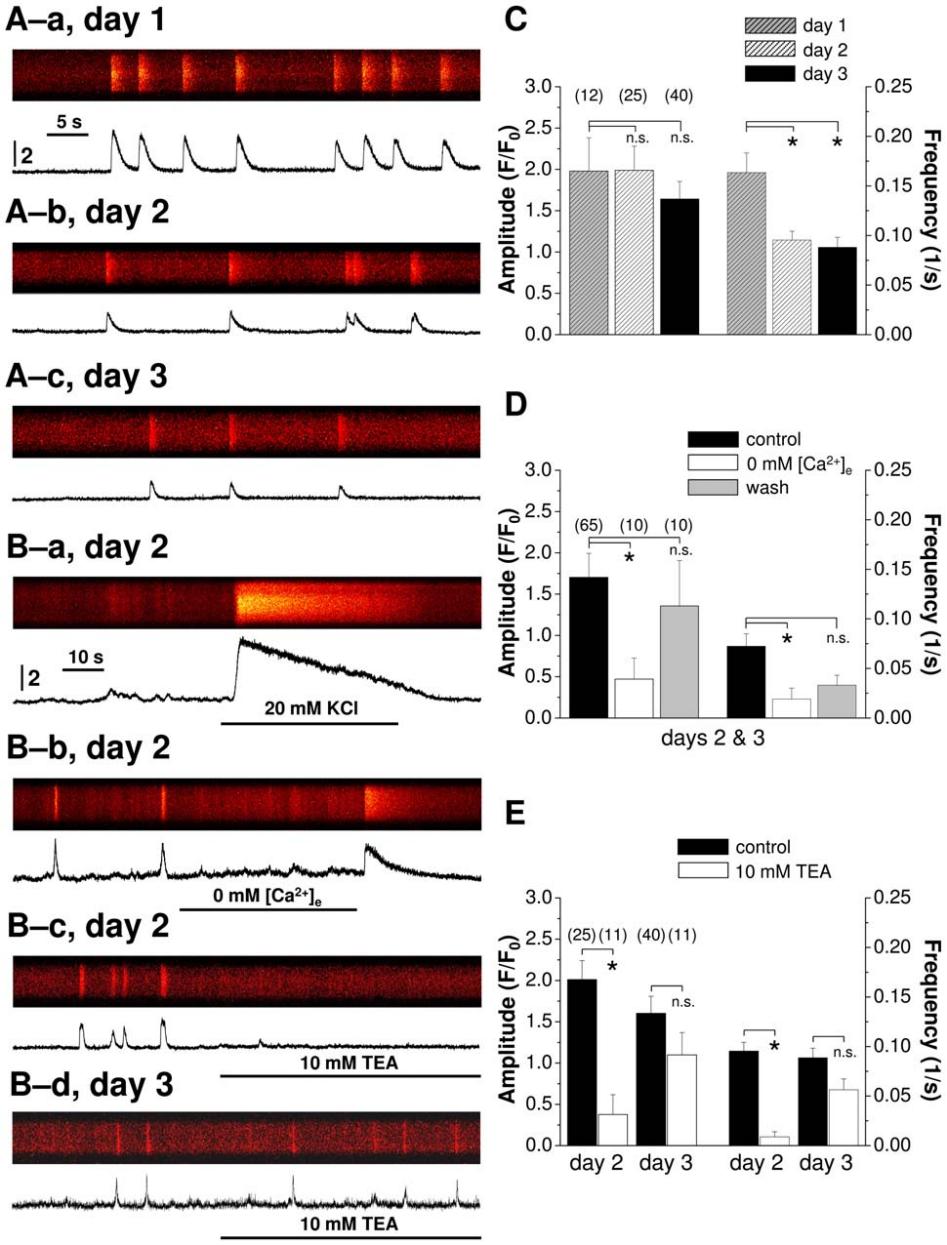
Spontaneous changes in [Ca^2+^]_i_ and effects of different ion composition and TEA on spontaneous calcium events in cells of HDC. (**A**) Representative line-scan images showing spontaneous changes in [Ca^2+^]_i_ on various days of culturing. Images were recorded at 7 ms/line, 512 pixels/line. (**B**) Representative line-scan images recorded on days 2 and 3 demonstrating the effects of the ion milieu or TEA application on spontaneous Ca^2+^ events. Images were acquired at 14 ms/line and 512 pixels/line. Horizontal lines indicate the application of different solutions. For panels (**A**) and (**B**), traces below the images are spatially averaged time courses of Fluo-4 fluorescence normalized to baseline fluorescence (F/F_0_). Vertical calibrations are the same for all images and for all traces in panels (**A**) and (**B**). (**C**) Pooled data for the amplitude and frequency of spontaneous calcium transients measured in normal [Ca^2+^]_e_ (1.8 mM) on different days of culturing. Asterisks (*) mark significant (p<0.05) differences between frequencies on different days of culturing as compared to day 1. Pooled data of the effects of (**D**) Ca^2+^-free milieu (days 2 and 3) and (**E**) 10 mM TEA (on day 2 and on day 3) on the amplitude and frequency of spontaneous calcium transients. Asterisks (*) indicate significant (p<0.05) differences between control and treated cells. Numbers above diagrams represent the number of cells measured during each measurement. Data represent mean ± standard error (SE) of the mean.

Differentiating chondrocytes in high density cultures (HDC) displayed rapid and repetitive [Ca^2+^]_i_ transients observed by confocal laser scanning microscopy during fluorescent calcium imaging (Fig. 1A), which resembled those characteristic of excitable cells. With differentiation, the frequency of the transients decreased, (0.163±0.020 Hz on day 1 and 0.089±0.010 Hz on day 3, ANOVA: *p*<0.01, Holm-Sidak (HS): *t* = 3.719) but their amplitude did not change significantly (expressed as F/F_0_: 1.98±0.40 on day 1 and 1.64±0.17 on day 3; ANOVA: *p*>0.05) where F and F_0_ are the fluorescence intensities of the peak and the baseline, respectively; Fig. 1Aa–c, Fig. 1C). The depolarization of the cell membrane by administration of 20 mM KCl on day 2 resulted in a large and transient increase in [Ca^2+^]_i_ (Fig. 1Ba). The rising phase of the KCl-evoked calcium transients was as rapid as that of the spontaneous transients, while [Ca^2+^]_i_ displayed a steady decline despite the continuous depolarization. Upon the removal of calcium from the external medium the amplitude and frequency of the repetitive calcium transients were decreased, sometimes completely eliminated (amplitude: to 0.47±0.25; ANOVA: *p* = 0.014, HS: *t* = 2.948 and frequency: to 0.019±0.011 Hz; ANOVA: *p*<0.01, HS: *t* = 3.341; Fig. 1Bb). The effect was reversible as evidenced from the pooled data presented in Fig. 1D. The addition of 10 mM TEA, a general blocker of voltage-gated K^+^ channels resulted in a significant decrease of both parameters of calcium oscillations on day 2 (amplitude: from 2.02±0.31 to 0.37±0.23; *p*<0.01, *F* = 10.74, Welch's correction, *t* = 4.164 and frequency: from 0.095±0.009 to 0.009±0.005 Hz; *p*<0.01, *F* = 19.38, Welch's correction, *t* = 8.268; Fig. 1Bc), but was non-significant on day 3 (amplitude: from 1.64±0.17 to 1.09±0.22, *p*>0.05, *F* = 3.392, *t* = 1.284 and frequency: 0.089±0.010 to 0.056±0.011 Hz; *p*>0.05, *F* = 2.998, *t* = 1.649; Figs. 1Bd and 1E).

### Patch-clamp experiments reveal changes in Na_V_ and K_V_ currents with the progression of chondrogenesis

The rapid nature of [Ca^2+^]_i_ oscillations and their sensitivity to external KCl and TEA prompted us to search for plasma membrane ion channels that may be involved in these phenomena via fast modification of the membrane potential.

Our strategy for the molecular identification of the channels was a multistep process, in which each step reduced the number of potential channel candidates. First we identified the charge carriers and the gating mechanisms of the currents. Then the thorough biophysical and pharmacological characterization further narrowed the possibilities based on V_1/2_ voltages, inactivation time constants and blocker IC_50_ values. Next, RT-PCR experiments were applied to eliminate some more candidates, before we carried out Western-blot experiments for final identification. Also the RT-PCR results gave us an idea about the possible time window of protein expression.

**Figure 2:**
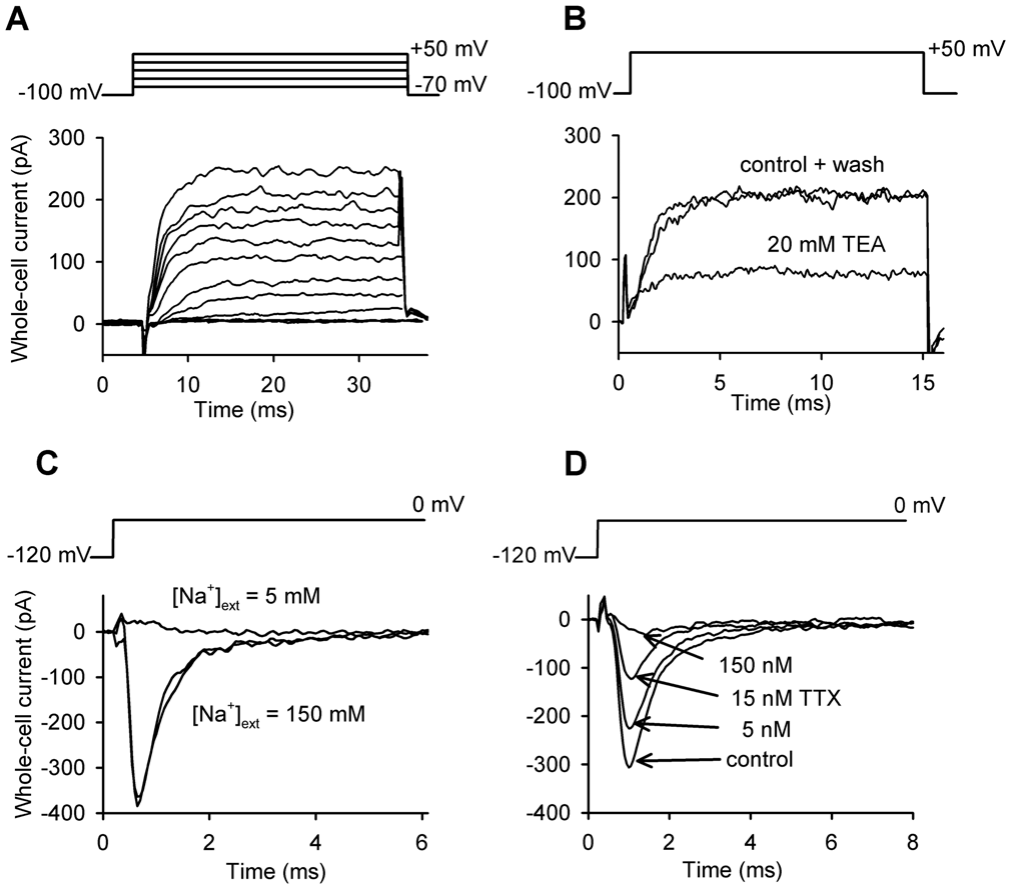
Electrophysiological properties of voltage-gated ion channels in chondrocytes. (**A**) Whole-cell K^+^ currents recorded from a patch-clamped chondrocyte one day after cell isolation. Depolarizing pulses ranging from −70 to +50 mV were applied every 15 seconds from a holding potential of −100 mV. Currents show voltage-dependent gating. (**B**) Application of 20 mM extracellular TEA reversibly blocked the K^+^ current. The cell was held at a holding potential of −100 mV and was depolarized to +50 mV every 15 s. (**C**) Whole-cell Na^+^ currents recorded from a patch-clamped chondrocyte two days after cell isolation. The cell was held at a holding potential of −120 mV and was depolarized to 0 mV every 15 s. The Na^+^ current disappeared in an external solution containing 5 mM Na^+^, identical to the Na^+^ concentration in the pipette. (**D**) Tetrodotoxin reversibly blocked the Na^+^ current in a dose dependent manner.

Using the whole-cell patch-clamp technique we found that depending on their differentiation state both inward and outward currents could be evoked by depolarizing pulses in chondrocytes (Fig. 2). The outward current detected at +50 mV was already present immediately after isolation (day 0) and remained detectable throughout the 3-day observation period in 60–80% of the cells, along with a gradual increase in the observed frequency of the inward current measured at 0 mV. This current was not seen within the first 24 hours following isolation, but its occurrence increased to 30% by day 1 and 70–75% by days 2 and 3. This suggests that the expression level of these channels can vary during differentiation and that they may have a role in the regulation of the process.

The activation of the outward current (Fig. 2A) required depolarizing pulses of −50 to −30 mV or more positive. It exhibited voltage dependence and its amplitude varied with changes in the external K^+^ concentration as would be expected for a K^+^ selective channel. The wide-spectrum K^+^ channel blocker TEA at 20 mM reversibly blocked the current (Fig. 2B), but its efficacy showed great cell-to-cell variation, which suggested that the total whole-cell K^+^ current was composed of several components. The different TEA sensitivity of the channels and the ratios of their numbers in the membrane could result in the heterogeneity of the TEA block, therefore, the calculated IC_50_≈7 mM of the pooled data (n = 33) may not be representative of a particular cell.

Figures 2C and D demonstrate the properties of the inward current activated by membrane depolarization. According to ion substitution experiments, the charge carrier proved to be Na^+^ (Fig. 2C) and the average Na^+^ current density was 68.5 pA/pF. This current also showed voltage-dependence, and tetrodotoxin (TTX), a high affinity blocker of several voltage-gated Na^+^ channels reversibly blocked the current supporting the assumption of Na_V_ channels being expressed in the membrane (Fig. 2D).

**Figure 3:**
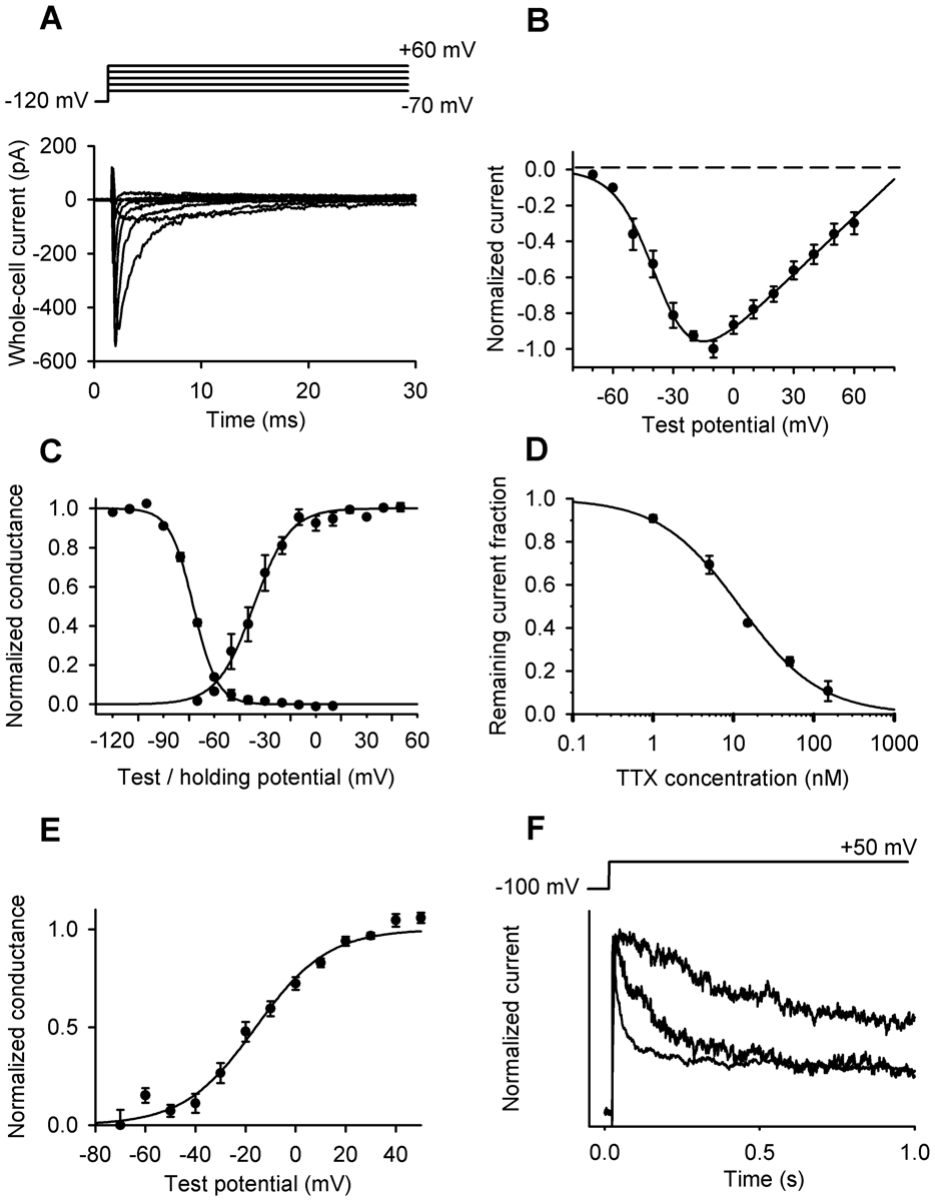
Detailed characterization of the voltage-gated Na^+^ and K^+^ currents. (**A**) Na^+^ currents evoked by depolarizing pulses from −70 to +60 mV in 10 mV increments in a 2-day-old patch-clamped differentiating chondrocyte. (**B**) The current–voltage relationship of the Na^+^ current determined from the peak current at each voltage. (**C**) The functions describing the voltage dependence of steady-state activation (G–V curve) and inactivation mark a potential window for Na^+^ channel operation. The G–V curve was constructed from the current–voltage relationship. The voltage dependence of steady-state inactivation was obtained by holding the cells at the indicated holding potentials for 15 s then the peak current was recorded during a pulse to 0 mV. Data points were fitted with Boltzmann-functions yielding the half-activation (V_1/2,a_ = −36.8 mV) and half-inactivation (V_1/2,i_ = −72.4 mV) voltages. (**D**) Dose–response function of tetrodotoxin on Na^+^ channels of differentiating chondrocytes. Fitting the Hill equation to the data points yielded K_d_ = 12 nM and n_H_ = 0.87. (**E**) Voltage dependence of the steady-state activation (G–V curve) of voltage-gated K^+^ channels in chondrocytes. The G–V relationship was constructed from the current–voltage relationship at each test potential using E_rev_ = −85 mV; points were fitted with a Boltzmann-function. The curve represents a mixture of K_V_1.1 and K_V_4.1 channels, and the obtained V_1/2_ = −15.5 mV lies between the V_1/2_ of those channels. (**F**) Normalized current traces recorded from three different cells representing the highly variable inactivation rate of the K^+^ current. Cells were depolarized to +50 mV for 1.5 s, the first 1 s is shown for clarity.

To get closer to the molecular identification of the channels a more detailed biophysical and pharmacological characterization was required, so further patch-clamp experiments utilizing various voltage-protocols were carried out. With a series of depolarizing pulses of increasing amplitude, the current-voltage relationship (I–V) for the Na^+^ conductance was obtained (Figs. 3A and B). From the I–V relationship the voltage-dependence of steady-state activation (G–V) curve was constructed and the half-activation voltage was determined from a Boltzmann-fit (V_1/2, a_ = −36.8 mV, Fig. 3C). Using the appropriate voltage-protocol (see figure legend) we also determined the voltage-dependence of steady-state inactivation (V_1/2,i_ = −72.4 mV). The overlay of these two curves revealed a potential window where the channels may be active (Fig. 3C). Finally, TTX affinity for the channel was determined by constructing the dose-response curve of the toxin, which yielded a K_d_ = 12 nM (Fig. 3D). This series of experiments reduced the number of candidates to a few types of TTX-sensitive voltage-gated Na^+^ channels.

The current-voltage relationship for the K^+^ conductance was also measured. However, similarly to TEA block, it showed considerable cell-to-cell variation strengthening the notion of more than one K^+^ channel being present in the membrane. Nevertheless, the data were pooled and the voltage-dependence of steady-state activation (G–V) curve was constructed (Fig. 3E, V_1/2_ = −15.5 mV). During long depolarizing pulses the K^+^ current inactivated and monoexponential fits yielded an average time constant of 316±40 ms (n = 27). Usually, however, inactivation kinetics was more complex and single exponentials gave a poor fit, again suggesting the simultaneous presence of more channel types or heteromeric channel assembly (Fig. 3F). Consequently, in a few cells extreme inactivation rates were seen and the steady-state current level also showed great variability.

### Chondrogenic cells express Na_V_1.4, K_V_1.1, K_V_1.3 and K_V_4.1 channels

**Figure 4:**
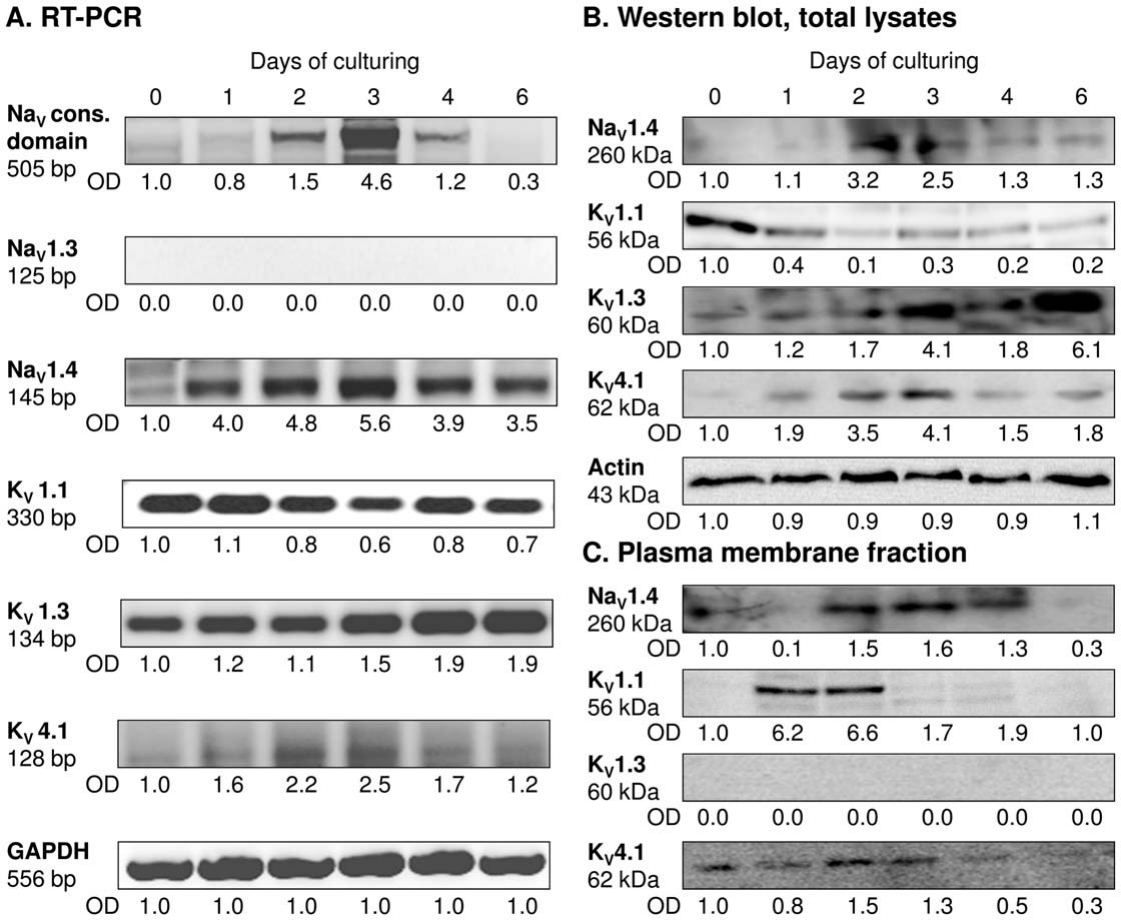
Expression profile of voltage-gated ion channels in cells of HDC on various days of culturing. mRNA (**A**) and protein (**B**) expression from total cell lysate of Na_V_1.4, K_V_1.1, K_V_1.3 and K_V_4.1in cells of HDC on various days of culturing. For RT-PCR reactions, GAPDH was used as a control. A degenerated Na_V_ primer was used as a positive control. (**C**) Membrane fraction samples were used to demonstrate the presence of channel proteins in the plasma membrane.

To identify the ion channels expressed in chicken mesenchymal cells during *in vitro* chondrogenic differentiation, the mRNA and protein expression pattern of Na_V_ and K_V_ channels were determined (Figs. 4A–B). RT-PCR experiments were first used to reduce the number of possible channels and avoid unnecessary Western-blots. Based on the biophysical and pharmacological properties of the Na^+^ current, Na_V_1.3 and 1.4 channels were the most likely candidates. As a positive control a degenerated primer pair of Na_V_ channels was used that amplifies cDNA for all known neuronal sodium channels in vertebrates [26]. mRNA expression of the Na_V_ conservative sequence in cells of HDC showed a peak-like pattern with a four-fold elevation on days 2 and 3 compared to day 0 (Fig. 4A). mRNA expression of Na_V_1.4 , but not of Na_V_1.3, was detected in cells of HDC by RT-PCR reactions on various days of culturing. Na_V_1.4 followed a pattern similar to the degenerated Na_V_ primer amplification (Fig. 4A).

The mRNA and protein expression profiles of K_V_ channels, likely to be expressed by HDC on the basis of their electrophysiological properties (TEA-sensitivity, voltage dependence (V_1/2_) and inactivation kinetics), were also studied. The mRNA expression level of K_V_1.3 was well detectable on days 0 and 1, then the signal became even stronger, while mRNA expression of K_V_4.1 was generally weaker and followed a peak-like pattern with a more than two-fold elevation on days 2 and 3 compared to day 0. To the contrary, the mRNA expression level of K_V_1.1 was found to be slightly higher on days 0 and 1 of culturing, then markedly decreased (Fig. 4A).

### K_V_1.1 expression in the plasma membrane coincides with the commitment of chondrogenic cells

**Figure 5:**
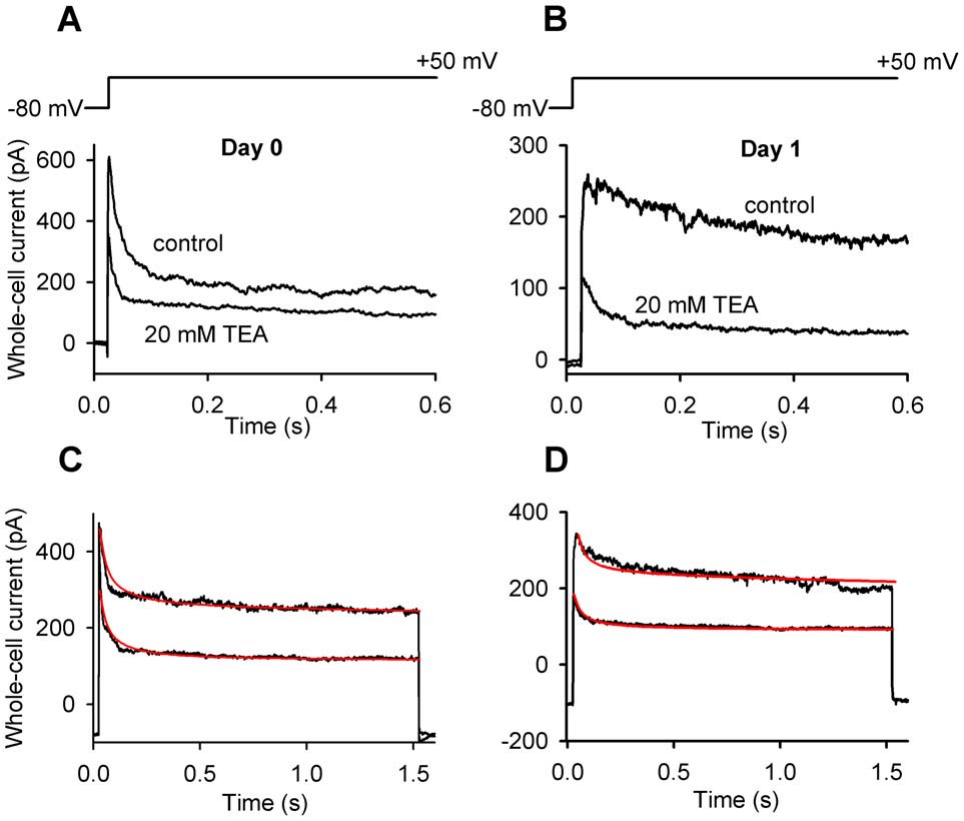
Changing kinetics and TEA-sensitivity of the K^+^ current during differentiation can be reproduced by simulated current traces. Whole-cell K^+^ currents recorded from a chondrogenic cell on the day of isolation (**A**) and on day 1 of culturing (**B**) in the absence and presence of 20 mM TEA. Currents were elicited by 1.5 s-long depolarizing pulses to +50 mV from a holding potential of −80 mV. For clarity the first 600 ms are shown. (**C and D**) Whole-cell K^+^ currents were modeled as the sum of currents through three different K^+^ channels: K_V_1.1, K_V_4.1 and a voltage-independent, possibly 2-pore channel. Simulations were based on the TEA sensitivity and inactivation kinetics. Calculated current traces with red lines are overlaid on recorded traces in the absence and presence of 20 mM TEA. The free parameters were the relative contributions of each channel type to the total whole-cell K^+^ current. Although more current components may also contribute, traces could be adequately reproduced by these three components. Traces on panels C and D are identical to those on panels A and B, respectively. The non-inactivating voltage-independent current component determined from the simulation was subtracted from the traces on panels A and B.

Western blots performed on total cell lysates were employed to establish whether the ion channel proteins were expressed by cells on various days of culturing. This, however, does not reflect on their functional expression in the plasma membrane. Therefore, to provide evidence on their plasma membrane expression patterns, it was necessary to perform Western blot analyses on protein samples isolated from the plasma membrane fraction of high density cultures. The total protein expression of the Na_V_ and K_V_ channels showed good correlation with their mRNA expression pattern (Fig. 4B). Under control conditions, the protein expression of Na_V_1.4 in cells of HDC exhibited a pattern that is closely correlated with the mRNA expression and reached its peak on days 2 and 3 with a three-fold elevation compared to day 0 (Fig. 4B). In the plasma membrane fraction, signals of Na_V_1.4 became markedly present from day 2 of culturing showing a peak-like pattern that closely coincides with the results observed in total cell lysates (Fig. 4C). After a high expression level on day 0, the protein expression of K_V_1.1 showed a constant signal in total cell lysates. While the protein expression of K_V_1.3 exhibited a rather variable pattern during chondrogenesis, K_V_4.1 protein expression followed a peak-like pattern and showed an almost four-fold elevation by days 2 and 3 compared to day 0, as revealed by Western blot analyses performed on total cell lysates (Fig. 4B). Presence of K_V_4.1 was well detectable in the plasma membrane during the entire culturing period, while K_V_1.1 exhibited a very prominent expression on days 1 and 2 of culturing. No plasma membrane signals were detected for K_V_1.3 channels (Fig. 4C). The changing expression level of K_V_1.1 and K_V_4.1 channels in the membrane was evident in the inactivation kinetics of the potassium current and its sensitivity to TEA (Fig. 5).

On the day of cell isolation, when K_V_1.1 expression is low, but K_V_4.1 channels are present in higher numbers, the whole-cell K^+^ current is dominated by fast-inactivating and TEA-insensitive K_V_4.1 channels, so 20 mM TEA causes only small reduction of the peak current (Fig. 5A). In contrast, on day 1 of culturing, when K_V_1.1 channels are strongly upregulated, the current is dominated by slowly inactivating and TEA-sensitive K_V_1.1 channels, so TEA application causes a more significant current reduction, and the smaller, fast-inactivating and TEA-insensitive component becomes apparent (Fig. 5B). These observations were confirmed by computer simulations of the changes in the kinetics and amplitude of current traces upon the application of TEA (Figs. 5C and 5D).

### Extracellular administration of TEA, but not that of TTX, decreases cartilage formation of HDC

**Figure 6:**
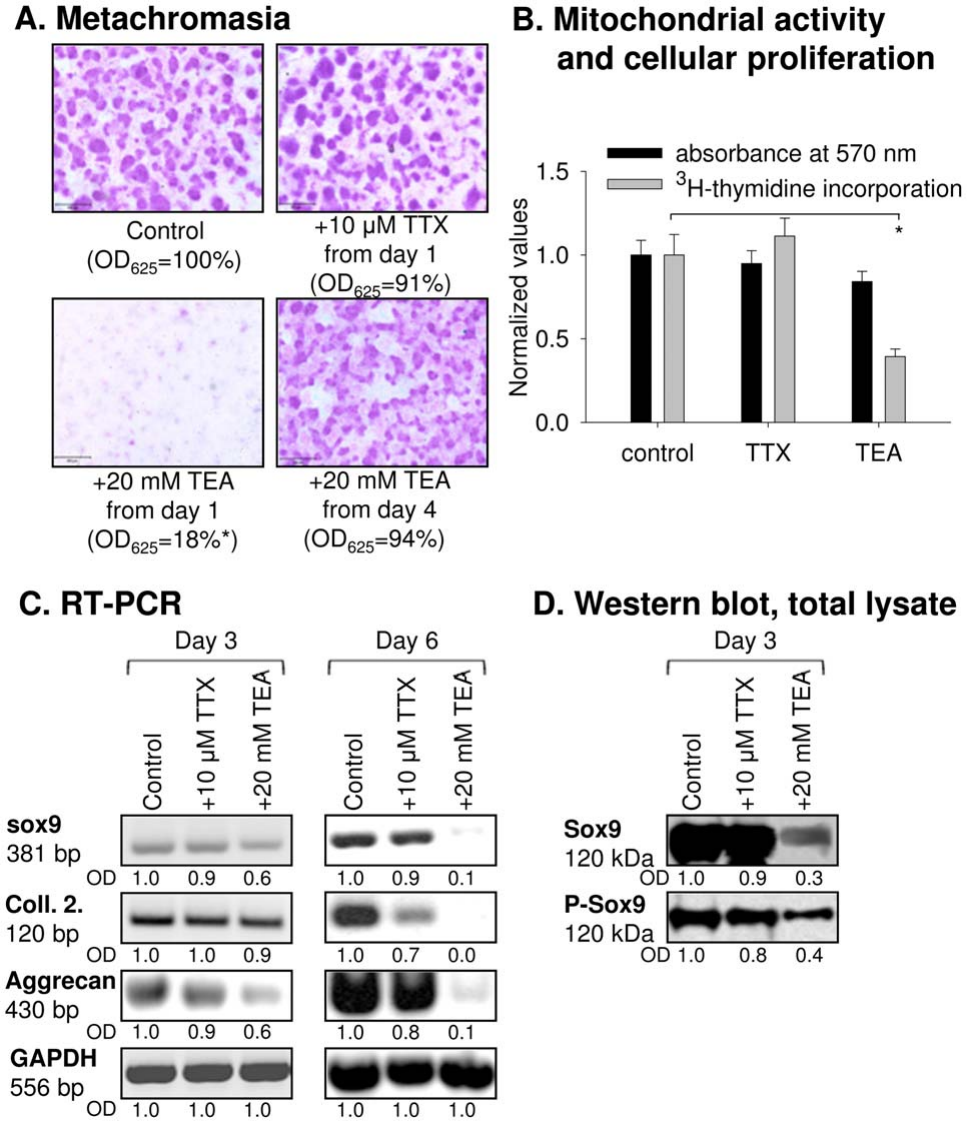
Effects of TTX (10 µM) and TEA (20 mM) on cartilage matrix production, viability and proliferation of HDC. (**A**) Metachromatic cartilage areas in 6-day-old high-density colonies were visualized with DMMB dissolved in 3% acetic acid. Optical density (OD_625_) was determined in samples containing toluidine blue extracted with 8% HCl dissolved in absolute ethanol. Scale bar, 500 µM. (**B**) Effects of TTX and TEA treatments on cellular metabolic activity and cellular proliferation in HDC. Assays were carried out during the administration of TTX and TEA. (**C–D**) Effects of TTX and TEA treatment on the mRNA expression of Sox9, type II collagen and aggrecan on days 3 and 6, and protein expression and phosphorylation level of Sox9 in HDC on day 3 of culturing. For RT-PCR reactions, GAPDH was used as a control.

Cartilage matrix production, as a direct indicator of chondrogenic differentiation, was analyzed by metachromatic staining with dimethyl-methylene blue (DMMB) and toluidine blue (TB) on day 6 of culturing. TTX did not cause significant changes in the metachromatic area of HDC (Fig. 6A). In sharp contrast, cartilage formation was dramatically decreased to approximately 20% of the control when TEA was administered from culture day 1. However, it remained unaffected when application of TEA was only started on day 4 (Fig. 6A).While the viability of chondrifying mesenchymal cells was not altered by either TEA or TTX (ANOVA, p>0.05), the proliferative ability of cells in HDC was significantly decreased by TEA, but not by TTX (ANOVA, p<0.01, HS: for TEA *t* = 4.391; for TTX, *t* = 0.813; Fig. 6B).

To confirm the results obtained with metachromatic staining, the mRNA expression of aggrecan and collagen type II was analyzed. Since cartilage formation was diminished by TEA, the mRNA and protein expression of the transcription factor Sox9, a positive regulator of chondrogenesis, was investigated as a possible downstream element responding to the inhibition of K^+^ channel activity. In accordance with matrix formation analyses, application of TTX did not cause considerable changes in the mRNA expression levels of aggrecan, collagen type II and Sox9 (Fig. 6C) or in the protein expression of Sox9 and its phosphorylated form (Fig. 6D). To the contrary, exposure to TEA resulted in a marked decrease in the mRNA expression of Sox9 by day 3, followed by a delayed reduction of the mRNA expression of aggrecan and collagen type II by day 6 (Fig. 6C). A significant decrease in the protein levels of both Sox9 and p-Sox9 was detected under the effect of TEA treatment (Fig. 6D). Lowering the concentration of either of the blockers had qualitatively the same effects on chondrogenesis as the higher concentration (Figs. S1 and S2).

### The membrane potential of differentiating chondrocytes is sensitive to TEA

**Figure 7:**
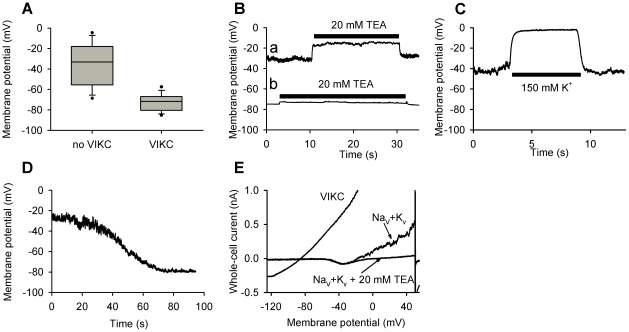
Membrane potential recordings from whole-cell patch-clamped chondrogenic cells. (**A**) The membrane potential distribution of the differentiating chondrogenic cells shows a bimodal distribution depending on the presence or absence of a voltage-independent K^+^ channel (VIKC). 5^th^, 10^th^, 25^th^, 50^th^, 75^th^, 90^th^ and 95^th^ percentiles are shown. (**B**) Application of 20 mM extracellular TEA caused a reversible depolarization of the cells, which was more significant for cells with more positive membrane potentials (**a**) than those with membrane potentials close to the K^+^ reversal potential (**b**). (**C**) Reversible depolarization of the membrane potential by the extracellular application of 150 K^+^. (**D**) Spontaneous hyperpolarization of the membrane potential of a patch-clamped chondrocyte close to the K^+^ reversal potential due to the activation of the voltage-independent K^+^ channel. (**E**) Spontaneous activation of the voltage-independent K^+^ channel during voltage-clamp recording. Whole-cell current traces were recorded during voltage-ramps running from −120 mV to +50 mV in 150 ms. Selected traces indicate the activity of the Na_V_ and K_V_ channels at the beginning of the recording, of which K_V_ channels were blocked by 20 mM TEA. During recording a large, voltage-independent and highly K^+^-selective conductance was activated as indicated by its reversal potential.

The inhibition of K^+^ channels by TEA is likely to influence the resting membrane potential of the cells. This scenario was tested by recording the membrane potential of chondrogenic cells in the current-clamp mode of patch-clamp on days 1–3. The membrane potential distribution of the cells was bimodal: in one population, the mean membrane potential was more positive and showed greater fluctuations with a large cell-to-cell variation (mean: −39.0±4.1 mV; standard deviation of fluctuations: 1.21±0.17 mV; n = 31). In contrast, the other population had far less “noisy” recordings, was much more hyperpolarized with significantly lower cell-to-cell variance (mean: −72.2±1.5 mV; standard deviation of fluctuations: 0.43±0.07 mV; n = 34, Figs. 7A and 7B). The membrane potential in both populations was highly K^+^-dependent since in symmetric K^+^ concentrations the cells were depolarized to 0.6±0.8 mV (Fig. 7C, n = 41) indicating the role of K^+^ channels in setting the membrane potential. In several cells a transition from the more positive value to the hyperpolarized membrane potential could be observed during recording suggesting the activation of a large, highly K^+^-selective conductance (Fig. 7D). Indeed, with voltage ramps we could detect a voltage-independent current in these cells, which transiently activated during recording and reversed at around −80 mV at physiological K^+^ concentrations (Fig. 7E). The current had properties similar to those of two-pore K^+^ channels, but this observation was not further investigated at this stage. Nevertheless, assuming the presence of this voltage-independent channel in the membrane along with the voltage-gated K_V_1.1 and K_V_4.1 channels at different ratios was necessary to adequately reproduce the recorded whole-cell K^+^ currents during various phases of differentiation (Figs. 5C and 5D).

The membrane potential of the more positive population was depolarized by 9.3±2.1 mV by 20 mM TEA (n = 10), while the membrane potential of the hyperpolarized population was less sensitive to TEA (depolarized by 3.8±1.4 mV, n = 19, *p* = 0.015, *F* = 1.285, *t* = −2.269) (Fig. 7B). Application of 100 nM TTX to cells on day 2 of culturing had no effect on the membrane potential suggesting that Na_V_ channels do not contribute significantly to the resting membrane potential (data not shown).

## Discussion

The expression level of plasma membrane channels often changes with the differentiation state of the cells, which in turn may influence further steps of the process [27]–[29]. Thus, identifying ion channels in differentiating chondrocytes and tracking the changes in their expression levels can lead to better understanding of the signaling steps during chondrogenesis. Several reports exist on the ion channels and membrane potential of mature chondrocytes, yet the available information is not consistent. Voltage-gated as well as Ca^2+^-activated potassium channels have been described in equine, elephant, canine and chicken chondrocytes with various biophysical and pharmacological properties [16], [17], [19], [21]–[23]. The membrane potential of canine chondrocytes was found to depend on the activity of a voltage-gated K^+^ channel, while that of rabbit chondrocytes was affected by a voltage-gated Cl^−^ channel, but was insensitive to K^+^ [19], [20]. The role of TRPV5 channels in setting a strongly depolarized resting potential was also suggested [30]. Classical ion channel blocking compounds modified the membrane potential and the matrix synthesis of chondrocytes [15], [18]. Other channels, including members of the TRP family, NMDA receptors, stretch-activated Ca^2+^ and K^+^ channels were implicated in mechanosensation and the regulation of chondrocyte proliferation and differentiation [31]–[35]. We report here for the first time that chondrifying mesenchymal cells display short-lived and high-frequency Ca^2+^ transients which are uncommon in non-excitable cells, along with the presence of voltage-gated Na^+^ and K^+^ channels in their plasma membranes. We found that the properties of the Ca^2+^ spikes, as well as the expression level of the ion channels changed during the first few days of differentiation when the most profound alterations in gene-expression, metabolism and morphology occur in the cells. We also demonstrated that the generation of Ca^2+^ transients, the maintenance of the membrane potential and cartilage matrix production all rely on the active function of K^+^ channels as revealed by the interference with these processes by the application of TEA. The fact that depolarization by raising external [K^+^] induced a Ca^2+^ transient further supports the role of voltage-gated channels in the Ca^2+^-signaling of these cells.

Based on our previous results indicating that calcium signaling is indispensable to proper differentiation of HDC we set out to identify plasma membrane ion channels that may be associated with this pathway. By using whole-cell patch-clamp, voltage-dependent outward and inward currents were detected, which proved to be K^+^ and Na^+^ conductances, respectively. The active role of plasma membrane channels during differentiation was demonstrated by their changing expression levels, which showed strong correlation with the progression of chondrogenesis. In line with the results of the patch-clamp measurements, the expression level of voltage-gated Na^+^ channels showed a gradual increase from their absence on the day of isolation to about 75% of the cells expressing it by day 3. These data were in complete agreement with the RT-PCR and Western blot results, which also demonstrated maximum expression of Na_V_1.4 on day 3 of culturing with the channels present in the membrane fraction as well. Nonetheless, pharmacological inhibition of this channel with TTX did not affect cartilage formation and TTX did not alter the resting membrane potential, which probably reflects on its accessory function in differentiating chondrocytes. Presence of Na_V_ channels in excitable cells is well documented and their fundamental role in the generation and propagation of action potential is widely accepted. Although several reports exist on the expression of Na_V_ channels by differentiated non-excitable cells, such as lymphocytes and macrophages [36]–[38], detecting them on differentiating chondrogenic cells is a novel and valuable finding, even though our experiments failed to unequivocally clarify the precise function of Na_V_ in these cells.

On the other hand, more data are available about the expression and possible function of K_V_ channels in non-excitable cells of very different nature. Normal epithelial cells from various organs, for example, express a great diversity of K_V_ channel subunits, and the role of K_V_ channels in the differentiation of immune cells, such as lymphocytes, macrophages and dendritic cells has been described by several groups [28], [29], [39]–[41]. The essential role of K_V_ channels in the regulation of cell proliferation in various normal and tumor cells is also discussed [42].

We observed the expression of K_V_1.1, K_V_1.3 and K_V_4.1 channels in cells of HDC. Patch-clamp measurements (days 0–3) were in complete agreement with these results and indicated that 60–80% of the cells displayed a voltage-gated K^+^ current, which period is characterized by an intense proliferation. Expansion of cell number is indispensable to proper chondrogenesis and indeed, blocking K_V_ activity with TEA resulted in the suppression of cell proliferation which, at least in part, can account for the inhibition of cartilage differentiation. Considering the facts that K_V_1.3 was undetectable in the plasma membrane fraction, and K_V_4.1 is virtually insensitive to TEA [43], the observed inhibition most likely have occurred via K_V_1.1 channels, which are highly sensitive to this compound (IC_50_≈0.3 mM, [44]). The role of K_V_ channels in the regulation of proliferation both in certain normal and malignant cells is widely documented [42], [45], implicating that K_V_1.3 is the primary voltage-gated K^+^ channel in these processes. Nonetheless, we were unable to detect this channel in the plasma membrane on any of the culturing days in spite of the protein being present in the whole cell lysates. This may indicate that K_V_1.3 channels were unable to reach the membrane possibly due to interference with their trafficking and were retained in the endoplasmic reticulum [46]. Alternatively, the K_V_1.3 signal may also come from its mitochondrial expression [47].

Changes of the membrane potential via modulation of the activity of K_V_ channels were described as an essential factor in the regulation of proliferation versus differentiation in human embryonic mesenchymal cells [48]. K_V_ channels have a fundamental role in adjusting the membrane potential, which in turn regulates the driving force of Ca^2+^ influx [49]. The latter is known to be a crucial factor in the regulation of cell proliferation [50]. Extremely high cell density, established via an initial rapid expansion of cell number is one of the prerequisites of proper chondrogenesis, and it probably requires a precisely set membrane potential adjusted to the demands of the steps of differentiation. Indeed, chondrogenic mesenchymal cells exhibited unique Ca^2+^ oscillations in our experiments, which were sensitive to the inhibition of the K^+^ channel inhibitor TEA. Many non-excitable cells respond to various stimuli by oscillatory Ca^2+^ transients (for a review, see [51]), however, spontaneous fluctuations of the [Ca^2+^]_i_ are only reported in case of a few cell types. Among them are human mesenchymal stem cells, in which [Ca^2+^]_i_ oscillations with an average frequency of 1 transient in 120 sec were reported [52]. In our experiments, however, cells of HDC exhibited spontaneous oscillations with an extraordinarily high frequency (approx. 5 in 60 sec on day 1), unlike any non-excitable cells reported earlier. The frequency of these oscillations gradually decreased as differentiation progressed, which is in accordance with the decline of the proliferation rate of chondrogenic cells. Indeed, one group of the investigated chondrogenic cells proved to be hyperpolarized during electrophysiological recordings, which cells might represent the cell population of HDC that entered the mitotic cycle. These observations are completely in agreement with results obtained on human embryonic stem cells, suggesting that membrane potential changes may act as a switch for cells: hyperpolarization favors entering the cell cycle, while depolarization facilitates differentiation [48]. Our observations are further supported by the fact that TEA treatments completely abolished Ca^2+^ oscillations on day 2, when K_V_1.1 expression in the plasma membrane is extremely prominent, proliferation is very intense and differentiation of chondroblasts commences. The opening threshold of K_V_1.1 is about 20 mV more negative than that of K_V_4.1 [44], [53]. The high plasma membrane expression of K_V_1.1 at the beginning of culturing, when cells are characterized by rapid proliferation that requires plasma membrane hyperpolarization [12] is in line with this electrophysiological difference between K_V_1.1 and K_V_4.1.

As chondrocytes proceed in their differentiation program, plasma membrane expression of K_V_1.1 decreases and cellular responses become less sensitive to the blockade of K_V_ channels by TEA: Ca^2+^ oscillations in 3-day-old cells were not significantly affected. Moreover, TEA did not alter cartilage formation when it was applied after the differentiation of chondrocytes (*i.e.* after day 4 of culturing), supporting the concept that K_V_1.1 channels play a crucial role in the proliferation and differentiation of chondrocytes, while differentiated cells use (the TEA-insensitive) K_V_4.1 to set their more depolarized membrane potential as they become postmitotic. The detailed relationship between the membrane potential changes and the [Ca^2+^]_i_ oscillations requires further analysis, keeping in mind that the inactivation gating of K_V_1.1 has been reported to be modulated by Ca^2+^ [54], implicating a mutually advantageous liaison of [Ca^2+^]_i_ oscillations and K_V_1.1 activity.

In summary, our results indicate that the K_V_1.1 channel seems to be essential in the control of membrane potential in differentiating chondrocytes, as its blockade results in membrane depolarization and negatively affects downstream events such as calcium signaling, Sox9 expression, proliferation and eventually, cartilage formation. These data on chondrogenic signaling may help improve *ex vivo* cell expansion and differentiation techniques used in cell-based cartilage regeneration therapies.

## Materials and Methods

### Cell culturing

Ross hybrid chicken embryos of Hamburger–Hamilton stages 22–24 were used to establish primary chondrifying high density mesenchymal cell cultures as described previously [10]. Briefly, distal parts of the limb buds of embryos were removed and 100 µL droplets of cell suspensions with a density of 1.5×10^7^ cells/mL were inoculated onto Petri dishes (Orange Scientifique, Braine-l'Alleud, Belgium). Day of inoculation was considered as day 0. Colonies were nourished with Ham's F12 medium (Sigma, St. Louis, MO, USA), supplemented with 10% fetal calf serum (Lonza Group Ltd., Basel, Switzerland) and were kept at 37°C in the presence of 5% CO_2_ and 80% humidity in a CO_2_ incubator. The medium was changed on every second day.

To exclude the possibility of performing measurements on muscle progenitor cells, 3-day-old cultures were immunostained for desmin and only a small population of cells having obvious elongated shape was positive. Cells used in this study and identified by their rounded shape never showed desmin positivity (data not shown).

### Pharmacological inhibition of Na_V_ and K_V_ channels

To modify the function/activity of voltage-gated Na^+^ and K^+^ channels, ion channel inhibitors were added to the culturing medium at various concentrations: 10 µM TTX (Alamone Labs, Jerusalem, Israel) for Na_V_ channels and 20 mM TEA (Sigma) for K_V_ channels were used in most experiments. The inhibitors were applied continuously from day 1 or in some cases from day 4 of culturing.

### Light microscopical morphology

For visualization of cartilage matrix production in HDC, low pH metachromatic staining was performed with DMMB (Aldrich, Germany) dissolved in 3% acetic acid on day 6 of culturing. HDC established from 30 µL droplets of the cell suspension of different experimental groups were cultured on round coverglasses (diameter: 10 mm; Menzel-Gläser, Menzel GmbH, Braunschweig, Germany) placed into 24-well culture plates. On day 6, cell cultures were fixed in a 4∶1 mixture of absolute ethanol and 40% formaldehyde, stained with 0.1% DMMB dissolved in 3% acetic acid, washed in acetic acid and mounted in gum arabic. Microphotographs of metachromatic cartilaginous nodules were taken with Spot Advanced camera on a Nikon Eclipse E800 microscope by using a 4× objective (numeric aperture: 0.13; Nikon Eclipse 800, Tokyo, Japan); image acquisition was performed by Spot Advanced software (version 4.6; Diagnostic Instruments, Inc., Burroughs, Sterling Heights, MI, USA). The amount of sulfated matrix components was determined with a semiquantitative method, by measuring the optical density of extracted TB (Reanal, Budapest, Hungary) bound to glycosaminoglycans in mature HDC as described previously [10]. Optical density was measured in samples from 3 cultures of each experimental group in 5 independent experiments.

### Confocal microscopy

Spontaneous calcium transients and the effects of different ionic milieu or TEA on the appearance of these events were monitored using LSM 510 META Laser Scanning Confocal Microscope (Zeiss, Oberkochen, Germany). Cells of HDC were incubated for 30 min at 37°C with 10 µM Fluo-4-AM in Ham's F12 medium. Calcium imaging was performed in normal (137 mM NaCl, 5.4 mM KCl, 0.5 mM MgCl_2_, 1.8 mM CaCl_2_, 11.8 mM HEPES, 1 g/L glucose; pH 7.4) Tyrode's solution. Calcium-free Tyrode's solution contained 5 mM EGTA, without addition of CaCl_2_. KCl solution was prepared from normal Tyrode's at 20 mM final concentration. TEA solution was prepared from normal Tyrode's at 10 mM final concentration. Line scan images were taken to monitor the fluorescence intensity during spontaneous activities. Data acquisition was performed by LSM 510 Software R4.0 (Zeiss). Images were recorded at 7 ms/line, or 14 ms/line, 512 pixels/line and 8192 lines using a 63× water immersion objective (numeric aperture: 1.2). Fluo-4-loaded cells were excited with a 488 nm argon ion laser and emitted fluorescence was collected at 500–570 nm. Images were analyzed using an automatic event detection program developed in the Department of Physiology, University of Debrecen.

### Cell proliferation and mitochondrial activity assays

Medium containing 1 µCi/mL ^3^H-thymidine (diluted from methyl-^3^H-thymidine; 185 GBq/mmol; Amersham Biosciences, Budapest, Hungary) was added to wells of opaque 96-well microtiter plates (Wallac, PerkinElmer Life and Analytical Sciences, Shelton, CT, USA) for 16 hrs on day 3 at the presence of voltage gated sodium and potassium channel blockers. After washing with PBS (phosphate buffered saline), proteins were precipitated with ice-cold 5% trichloroacetic acid, and washed with PBS again. Colonies were then air-dried for 1 week and radioactivity was counted by Chameleon liquid scintillation counter (Chameleon, Hidex Ltd., Turku, Finland). Measurements were carried out in 10 samples of each experimental group in 5 independent experiments.

For investigation of mitochondrial activity, cells cultured in 96-well plates were used. 10 µL MTT reagent [3-(4,5-dimethylthiazolyl-2)-2,5-diphenyltetrazolium bromide; 5 mg MTT/1 mL PBS] was pipetted into each well. Cells were incubated for 2 hours at 37°C and following addition of 500 µL of MTT solubilizing solution (10% Triton X-100 and 0.1 M HCl dissolved in isopropanol), optical density was measured at 570 nm (Chameleon, Hidex Ltd.).

### RT-PCR analysis

Cell cultures were dissolved in Trizol (Applied Biosystems, Foster City, CA, USA) and after the addition of 20% RNase free chloroform, samples were centrifuged at 4°C at 10,000×*g* for 15 min. Samples were incubated in 500 µL of RNase free isopropanol in −20°C for 1 h, then total RNA was harvested in RNase free water and stored at −20°C. The assay mixture for reverse transcriptase reaction contained 2 µg RNA, 0.112 µM oligo(dT), 0.5 mM dNTP, 200 units of High Capacity RT (Applied Bio-Systems) in 1× RT buffer. The sequence of degenerated primer pairs was predicted to amplify cDNA for all known neuronal sodium channels in vertebrates [26]. The sequence was: 5′–GAT YCT SCT CAG YAG TGG–3′, and 5′–CAT RAT RTC CAT CCA KCC–3′. The sequences of chicken specific primer pairs were as follows: for chicken aggrecan (Accession No.: XM_001232949) 5′–CAA TGC AGA GTA CAG AGA–3′ and 5′–TCT GTC TCA CGG ACA CCG–3′; for chicken Sox9 (Accession No.: AB012236) 5′–CCC CAA CGC CAT CTT CAA–3′ and 5′–CTG CTG ATG CCG TAG GTA–3′; for chicken collagen type II (Accession No.: NM_204426) 5′–CCC TCA AAT CCC TCA ACA ATC A–3′ and 5′–AAT CTC GCT CTT CCA CTC G–3′ and for chicken GAPDH (Accession No.: NM_204305) 5′–GAG AAC GGG AAA CTT GTC AT–3′ and 5′–GGC AGG TCA GGT CAA CAA–3′. The sequences of primer pairs designed to amplify the conservative domain of human voltage gated sodium and potassium channels were as follows: for Na_V_1.3 (Accession No.: NM_013119) 5′–GAC TCA TGA CTC AGG ACT A–3′ and 5′–GAT CAA GTT CAC CAA ATA A–3′; for Na_V_1.4 (Accession No.: NM_013178) 5′–CCG AGG ATG AGA AGA AGG–3′ and 5′–ATG GTG AGG TAG GGG TTG–3′; for K_V_1.1 (Accession No.: NM_000217) 5′–AAA CAT TGC TGG ACT ACG C –3′ and 5′–CGC TGG TAT TCT CCC TCT–3′; for K_V_1.3 (Accession No.: NM_002232) 5′–AGC CGA GAA CCA GCA GAG–3′ and 5′–GAC GAG GTG GCA TTG AG–3′; and for K_V_4.1 (Accession No.: NM_002233) 5′–TGG AAG AAT ACG CTG GAC C–3′ and 5′–CAA GGC GGT ACA CGA CTT–3′.

Amplifications were performed in a thermocycler as follows: 94°C, 1 min, followed by 30 cycles (94°C, 30 sec, 54°C, 30 sec, 72°C, 30 sec) and then 72°C, 5 min. After adding 1/5 volume of fivefold concentrated DNA sample buffer (0.41% bromophenol blue, 66.6% sucrose in TAE buffer containing 0.016 M EDTA, 0.19 M acetic acid and 0.4 M Tris–HCl; pH 8.5) PCR products were analyzed by electrophoresis in 1.2% agarose gel containing ethidium bromide. Optical density of signals was measured by using ImageJ 1.40 g freeware and results were normalized to the optical density of untreated control cultures.

### Preparation of cell extracts

Three-day-old cell cultures were washed in physiological NaCl solution and were harvested. After centrifugation cell pellets were suspended in 100 µL of homogenization buffer containing 50 mM Tris–HCl buffer (pH 7.0), 10 µg/mL Gordox, 10 µg/mL leupeptine, 1 mM phenylmethylsulphonyl-fluoride (PMSF), 5 mM benzamidine, 10 µg/mL trypsin inhibitor as protease inhibitors. Samples were stored at −70°C. Suspensions were sonicated by pulsing burst for 30 sec on 40 A (Cole-Parmer, Illinois, USA). For Western blotting, total cell lysates were used. For isolation of plasma membrane fraction, sonicated samples were centrifuged at 50,000×*g* for 90 min at 4°C. Pellet was extracted in 50 µL homogenization buffer supplemented with 1% Triton X-100 at 4°C for 1 h, then centrifuged again at 50,000×*g* for 55 min at 4°C and supernatant containing plasma membrane fraction was used for Western blot analyses.

### Western blot analysis

Samples for SDS-PAGE were prepared by the addition of fivefold concentrated electrophoresis sample buffer (20 mM Tris–HCl pH 7.4, 0.01% bromophenol blue dissolved in 10% SDS, 100 mM β-mercaptoethanol) to cell lysates to set equal protein concentration of samples, and boiled for 10 min. About 70–80 µg of protein was separated by 10% SDS-PAGE gel for detection of Sox9, p-Sox9, Na_V_1.4, K_V_1.1, K_V_1.3 and K_V_4.1. Proteins were transferred electrophoretically to nitrocellulose membranes. After blocking in 5% non-fat dry milk in PBST (phosphate buffered saline with 0.1% Tween 20, 20 mM Na_2_HPO_4_, 115 mM NaCl; pH 7.4), the membranes were washed and exposed to the primary antibodies overnight at 4°C as follows: polyclonal anti-Sox9 (Abcam, Cambridge, UK) in 1∶600, polyclonal anti-p-Sox9 (Sigma) in 1∶600, polyclonal anti-K_V_1.1 in 1∶200 (Abcam), polyclonal anti-Na_V_1.4 in 1∶200, polyclonal anti-K_V_1.3 in 1∶200 and polyclonal anti-K_V_4.1 antibody in 1∶400 dilution (all from Alamone Labs, Jerusalem, Israel) were used. After washing for 30 minutes in PBST, membranes were incubated with the secondary antibody, anti-rabbit IgG (Bio-Rad Laboratories, CA, USA) in 1∶1500 dilution. Signals were detected by enhanced chemiluminescence (Millipore, Billerica, MA, USA) according to the instructions of the manufacturer.

### Electrophysiology

For the measurement of ionic currents and membrane potentials, standard whole-cell patch-clamp procedures were performed using a Na^+^-based extracellular and a K^+^-based intracellular solution supplemented with 5 mM Na^+^. Whole-cell measurements were performed using Axopatch 200A and 200 B amplifiers connected to Axon Digidata 1200 and 1322A data acquisition hardware (Molecular Devices, Sunnyvale, CA). Pipettes were pulled from GC 150 F-15 borosilicate glass resulting in electrodes having 3- to 5-MΩ resistance in the bath. The bath solution consisted of 145 mM NaCl, 5 mM KCl, 1 mM MgCl_2_, 2.5 mM CaCl_2_, 5.5 mM glucose, and 10 mM HEPES; pH 7.35. The internal solution consisted of 140 mM KF or KCl, 5 mM NaCl, 2 mM MgCl_2_, 1 mM CaCl_2_, 10 mM HEPES, and 11 mM EGTA; pH 7.22. For data acquisition and analysis, the pClamp9/10 software package (Molecular Devices) was used. Before analysis, current traces were corrected for ohmic leak and digitally filtered (three-point boxcar smoothing). Each data point on the steady-state activation, inactivation and dose-response curve represents the mean of 3–14 independent experiments, and error bars represent standard error of the mean. Data points on the dose-response curve were fitted with a two parameter Hill-equation: RCF = K_d_^H^/(K_d_^H^+[Tx]^H^), where RCF is the Remaining Current Fraction, K_d_ is the dissociation constant, H is the Hill-coefficient and [Tx] is the toxin concentration.

Due to the very different kinetic parameters of the K_V_ and Na_V_ channels we did not apply pharmacological isolation of the two currents, rather, we took advantage of the difference in the biophysical parameters of gating (i.e., biophysical separation). The Na^+^ current reaches its peak and almost fully inactivates within 2 ms from the start of the depolarizing pulse even at 0 mV, while the K^+^ current reaches its peak value only about 4–5 ms after the start of the pulse at +50 mV. At +50 mV the Na^+^ current activates even faster; furthermore, the driving force for Na^+^ at this voltage is quite small, therefore the Na^+^ current present during the measurement of K^+^ currents has negligible amplitude and decays completely by the time the K^+^ current reaches its peak. Similarly, at 0 mV, where Na^+^ currents were measured, K^+^ currents have much slower activation kinetics and reduced amplitude. Nevertheless, when current records were visibly contaminated, e.g. by the voltage-independent K^+^ current, they were omitted from the analysis.

### Modeling of whole-cell potassium currents

Whole-cell K^+^ currents were modeled in Microsoft Excel as the sum of currents through three different K^+^ channels: K_V_1.1, K_V_4.1 and a voltage-independent, possibly 2-pore channel. Simulations were based on the TEA sensitivity and inactivation kinetics. Current traces were calculated in the absence and presence of 20 mM TEA with the free parameters being the relative contributions of each channel type to the total whole-cell K^+^ current. The applied parameters for TEA IC_50_ and inactivation time constants were as follows: K_V_1.1: 0.3 mM and 4.3 s; K_V_4.1: 150 mM and three components 25 (20%), 80 (40%) and 250 ms (40%), and the voltage-independent current: 20 mM and no inactivation.

### Statistical Analysis

All data are representative of at least five different experiments. Averages are expressed as mean ± standard error of the mean (*n*, number of independent experiments). For pairwise comparisons Student's *t*-test, for multiple comparisons one-way ANOVA with post-hoc Holm-Sidak (HS) test were used. * represents a significant difference, p<0.05.

## Supporting Information

Figure S1 **Effects of TTX (60 nM) on cartilage matrix production of HDC.** (**A**) 60 nM TTX was applied continuously from day 1. Metachromatic cartilage areas in 6-day-old high-density colonies were visualized with DMMB dissolved in 3% acetic acid. Optical density (OD_625_) was determined in samples containing toluidine blue extracted with 8% HCl dissolved in absolute ethanol. Scale bar, 500 µM. (**B–C**) Effects of TTX treatment on the mRNA expression of Sox9, type II collagen and aggrecan on day 3, and protein expression and phosphorylation level of Sox9 in HDC on day 3 of culturing. For RT-PCR reactions, GAPDH was used as a control.
(TIF)

Figure S2 **Effects of TEA (2 mM) on cartilage matrix production and proliferation of HDC.** (**A**) 2 mM TEA was applied continuously from day 1. Metachromatic cartilage areas in 6-day-old high-density colonies were visualized with DMMB dissolved in 3% acetic acid. Optical density (OD_625_) was determined in samples containing toluidine blue extracted with 8% HCl dissolved in absolute ethanol. Scale bar, 500 µM. (**B–C**) Effects of TEA treatment on the mRNA expression of Sox9, type II collagen and aggrecan on day 3, and protein expression and phosphorylation level of Sox9 in HDC on day 3 of culturing. For RT-PCR reactions, GAPDH was used as a control. (**D**) Effects of TEA treatments on cellular proliferation in HDC. Assays were carried out during the administration of TEA.
(TIF)
