# Supplementary figures and images for: Switch of Voltage-Gated K+ Channel Expression in the Plasma Membrane of Chondrogenic Cells Affects Cytosolic Ca2+-Oscillations and Cartilage Formation
Source: PLoS One. 2011 Nov 21;6(11):e27957. doi: 10.1371/journal.pone.0027957 (PMC3221679; doi:10.1371/journal.pone.0027957)

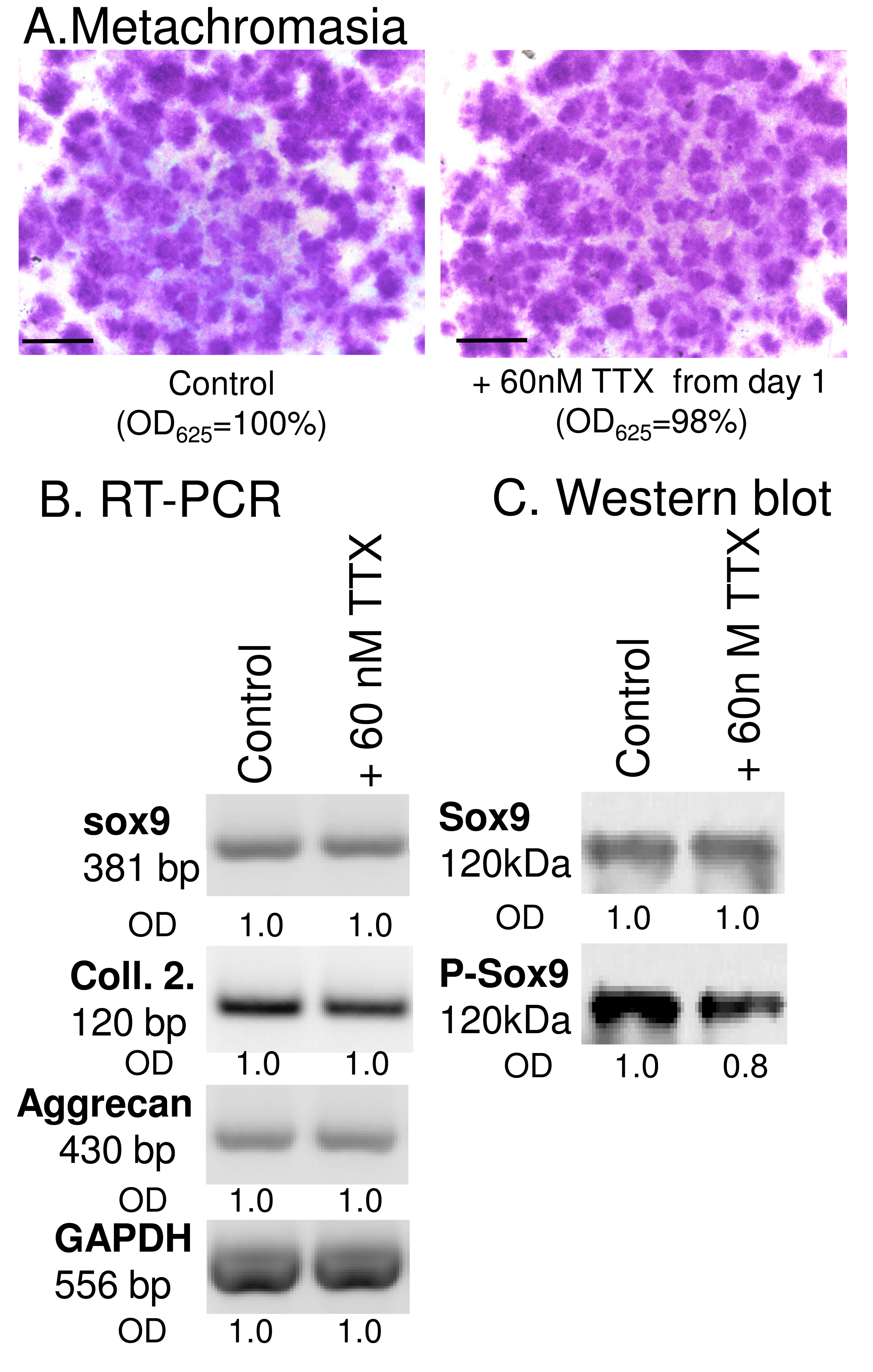

Supplement: Figure S1 — Effects of TTX (60 nM) on cartilage matrix production of HDC. (A) 60 nM TTX was applied continuously from day 1. Metachromatic cartilage areas in 6-day-old high-density colonies were visualized with DMMB dissolved in 3% acetic acid. Optical density (OD625) was determined in samples containing toluidine blue extracted with 8% HCl dissolved in absolute ethanol. Scale bar, 500 µM. (B–C) Effects of TTX treatment on the mRNA expression of Sox9, type II collagen and aggrecan on day 3, and protein expression and phosphorylation level of Sox9 in HDC on day 3 of culturing. For RT-PCR reactions, GAPDH was used as a control. (TIF) [file pone.0027957.s001.tif]

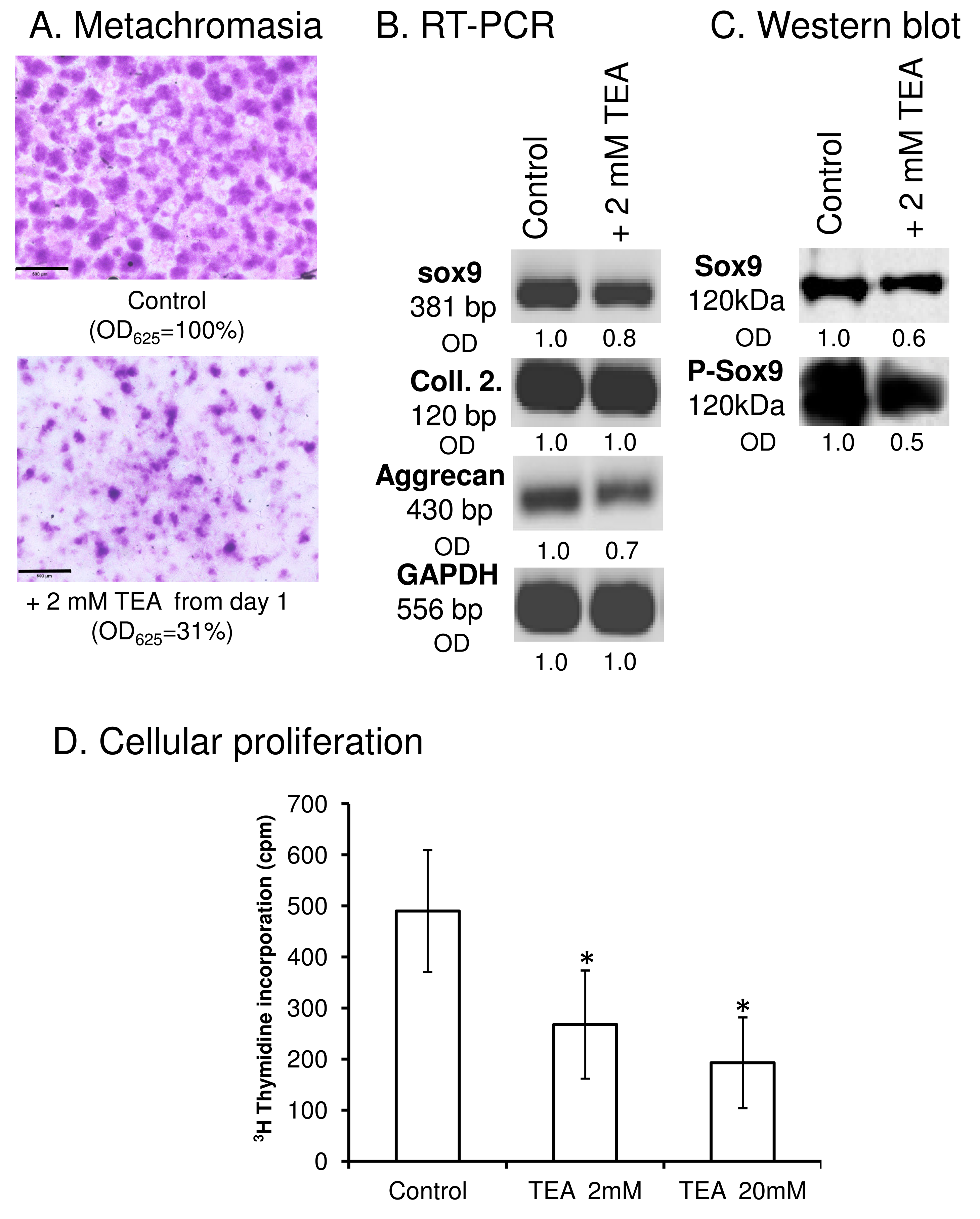

Supplement: Figure S2 — Effects of TEA (2 mM) on cartilage matrix production and proliferation of HDC. (A) 2 mM TEA was applied continuously from day 1. Metachromatic cartilage areas in 6-day-old high-density colonies were visualized with DMMB dissolved in 3% acetic acid. Optical density (OD625) was determined in samples containing toluidine blue extracted with 8% HCl dissolved in absolute ethanol. Scale bar, 500 µM. (B–C) Effects of TEA treatment on the mRNA expression of Sox9, type II collagen and aggrecan on day 3, and protein expression and phosphorylation level of Sox9 in HDC on day 3 of culturing. For RT-PCR reactions, GAPDH was used as a control. (D) Effects of TEA treatments on cellular proliferation in HDC. Assays were carried out during the administration of TEA. (TIF) [file pone.0027957.s002.tif]
